# An Unusual Cause of Cervical Radicular Pain-Foreign Body in Esophagus

**Published:** 2018-07

**Authors:** Edmond-Jonathan Gandham, Amit Tyagi, Krishna Prabhu

**Affiliations:** 1 *Department of Neurological Sciences, Christian Medical College, Vellore, India.*; 2 *Department of Otolaryngology, Christian Medical College, Vellore, India.*

**Keywords:** Esophagus, Foreign body, Radicular pain, Vertebral artery.

## Abstract

**Introduction:**

Foreign bodies in the esophagus are considered to be a life-threatening condition in adults and children because of esophageal perforation, chemical pneumonitis, airway obstruction, and development of a fistula, leading to high morbidity and mortality with this condition. Most cases present with immediate symptoms. However, in rare cases, the foreign body can migrate within the tissues and become symptomatic at a later date.

**Case Report:**

We report a rare case of a foreign body in the esophagus following fishmeal ingestion. The foreign body had traversed the lumen of the esophagus and migrated into the neural foramina with impingement of the left C6 root with resulting left C6 radicular pain. Radiology and successful surgical management is discussed herein, along with relevant literature.

**Conclusion:**

Radiculopathy after foreign body ingestion is very rare. In patients presenting with persistent radicular pain, in particular in close proximity to the neurovascular structures, we advise early surgery to prevent a neurological deficit.

## Introduction

Chicken and fish bones are the most frequently ingested foreign bodies and are commonly seen in the elderly and pediatric age groups. The tonsils, base of the tongue, and upper esophagus are common places for impaction (1). Removal of foreign bodies is essential to prevent infection, life-threatening complications such as perforation, and chemical mediastinitis.

## Case Report

A 54-year-old female was referred from the emergency department with sudden onset of dysphagia and odynophagia after ingestion of fishmeal. This was accompanied by a severe left C6 radicular pain. On examination, the patient had no focal neurological deficits.

**Fig 1:**
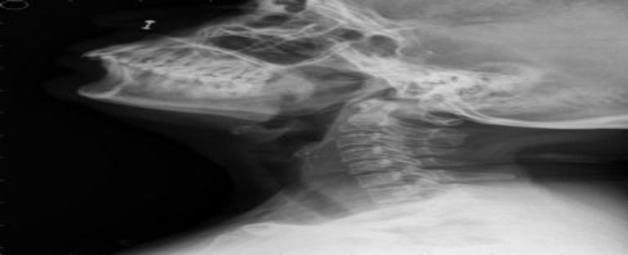
Lateral X-ray of the neck showing a radiopaque ingested foreign body, 3.5 cm, in the upper cervical esophagus with the sharp end in the neural foramen at the C5-6 level

A lateral X-ray of the neck showed a radiopaque foreign body, 3.5 cm in size in the mediolateral dimension, located in the upper cervical esophagus with the sharp end in the neural foramen at the C5-6 level (Fig.1). 

**Fig 2:**
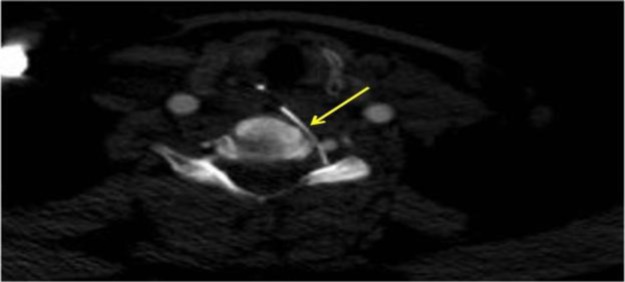
CT angiogram of the cervical spine showing transluminal migration of the foreign body through the esophagus, and its close relation to the left vertebral artery. The foreign body is depicted with a yellow arrow

A computed tomography (CT) angiogram of the cervical spine revealed transluminal migration of the foreign body across the esophagus into the left C5-6 neural foramen abutting the left vertebral artery. There was no evident vertebral artery injury (Fig.2).

**Fig 3:**
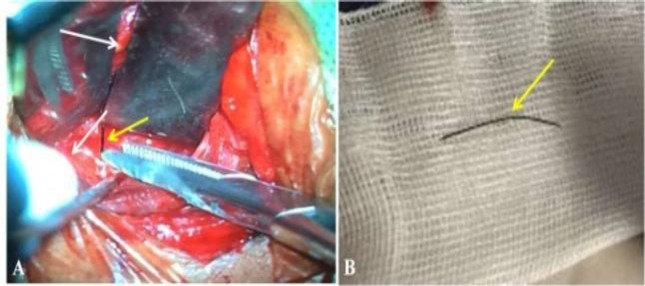
A) Intraoperative picture depicting the foreign body; the small arrow depicts the carotids, and the large arrow depicts the trachea-esophageal groove. The foreign body is depicted with a yellow arrow; B) Metallic foreign body measuring 3.5 cm. The foreign body is depicted with a yellow arrow

In view of the proximity to the neurovascular structures, the otolaryngologists deferred endoscopic removal of the foreign body. We approached the lesion through an anterior cervical approach with a standard incision used for anterior cervical discectomy on the left side. After retraction of the sternocleidomastoid and the carotid laterally, a hard-metallic linear foreign body was encountered traversing the lumen of the esophagus. The fascial dissection was carried out until the object was clear. The longus colli muscle and the C5-6 disc space was exposed. The foreign body was seen entering into the left C5-6 neural foramen. The metallic body was gently pulled and extracted completely with no injury to the vertebral artery injury (Fig.3). The patient had immediate relief of her left radicular pain; she was managed with Ryle’s tube feeds for a week and subsequently started on oral feeds and discharged.

## Discussion

The majority of patients with foreign body ingestion belong to the pediatric age group, although adults in specific risk groups such as prisoners, alcoholics, elderly adults with dentures, psychiatric patients, and those suffering from esophageal strictures can also be prone to foreign body ingestion (2). In our case, there were no adult risk factors. The most frequently ingested foreign bodies include chicken and fish bones in adults (1). A foreign body in the esophagus (FBE) is commonly located at one of the three esophageal rings; namely, the cricopharyngeal ring, the aortic arch narrowing, or at the esophageal-gastric junction. The FBE was located at the upper cervical esophagus in our patient. The location of the foreign body differs with age and is commonly impacted at the lower esophagus in adults (2,3).

The most common presentations are dysphagia, odynophagia, diffuse chest pain, laryngeal irritation, cough, and incomplete airway obstruction (4). An esophageal foreign body presenting with radicular pain is very rare, although there are few reports of penetrating neck injuries presenting with spinal cord injury (5). The incidence of vertebral artery injury (VAI) in the penetrating neck injury ranges from 1.0% for gunshot injuries to 7.4% for stab injuries (5).

Accurate imaging is required to locate the foreign body. X-ray and CT scans help in localizing the FBE, its trajectory, fragments in the spinal canal and proximity to the critical neurovascular structures (6). We performed a CT angiography as the FBE was located in the neural foramen, and there was no evidence of vertebral artery injury. Magnetic resonance imaging (MRI) is not indicated for metallic FBE due to artifacts.

The choice of treatment for foreign body removal depends on many factors such as patient age, clinical condition, size and shape of the foreign body, and its anatomic location (7). Endoscopy is the most commonly used method for extraction (7), with the advantages being direct visualization and ease of extraction, and evaluation of the degree of the injury. Rigid and flexible endoscopy is safe and effective in experienced hands. The morbidity rate with endoscopy is less than 1% (7). In our case, endoscopy was not attempted due to the proximity to the neurovascular structures. Surgery is considered, as in our case, when there is evidence of perforation, proximity to the neurovascular structures, or when not amenable for endoscopic removal. Surgical extraction of foreign bodies in the spinal canal is mandatory to prevent infection, myelopathy, and radiculopathy.

## Conclusion

Radiculopathy after foreign body ingestion is very rare. In patients presenting with persistent radicular pain and those which are in proximity to the neurovascular structures, we advise early surgery to prevent a neurological deficit.

## References

[1] Chee LW, Sethi DS (1999). Diagnostic and therapeutic approach to migrating foreign bodies. Ann Otol Rhinol Laryngol.

[2] Pelucchi S, Bianchini C, Ciorba A, Pastore A (2007). Unusual foreign body in the upper cervical oesophagus: case report. Acta Otorhinolaryngol Ital.

[3] Al-Qudah A, Daradkeh S, Abu-Khalaf M (1998). Esophageal foreign bodies. Eur J Cardiothoracic Surg.

[4] Chadha SK, Gopalakrishnan S, Gopinath N (2003). An unusual sharp foreign body in the esophagus and its removal. Otolaryngol Head Neck Surg.

[5] Xia X, Zhang F, Lu F, Jiang J, Wang L, Ma X (2012). Stab wound with lodged knife tip causing spinal cord and vertebral artery injuries: case report and literature review. Spine.

[6] De Lucas EM, Ruiz-Delgado ML, Garcia-Baron PL, Sadaba P, Pagola MA (2004). Foreign esophageal body impaction: multimodality imaging diagnosis. Emerg Radiol.

[7] Athanassiadi K, Gerazounis M, Metaxas E, Kalantzi N (2002). Management of esophageal foreign bodies: a retrospective review of 400 cases. Eur J Cardiothorac Surg..

